# Human herpesvirus 6 infection as a trigger of multiple sclerosis: an update of recent literature

**DOI:** 10.1186/s12883-022-02568-7

**Published:** 2022-02-15

**Authors:** K. I. Voumvourakis, P.C. Fragkou, D. K. Kitsos, K. Foska, M. Chondrogianni, S. Tsiodras

**Affiliations:** 1 2nd Department of Neurology, Attikon University Hospital, National and Kapodistrian University of Athens, Athens, Greece; 2 4th Department of Internal Medicine, Attikon University Hospital, National and Kapodistrian University of Athens, Athens, Greece

**Keywords:** Cerebrospinal fluid, CSF, Demyelination, Diagnosis, HHV-6, Human herpes virus 6,IgG antibodies, IgM antibodies, Multiple sclerosis, Pathogenesis,pcr, Polymerase Chain Reaction, Polymorphisms, Serology, Etiology

## Abstract

**Background:**

This is an update on the existing evidence regarding a relationship between infection with human herpesvirus 6 (HHV-6) and multiple sclerosis (MS) in order to contribute on the attempt to define the nature and strength of that relationship.

**Results:**

Study quality was assessed using the criteria proposed by Moore and Wolfson and by the classification criteria used by the Canadian Task Force on the Periodic Health Examination. Studies were categorized both by experimental technique and by quality (high [A], intermediate [B], and low [C]) as determined by the Moore and Wolfson criteria. Overall, 27 (90%) of 30 studies, 18 (86%) of which were classified as A quality, reached a statistically significant result. According to the Canadian Task Force classification, all studies were categorized as evidence of qualityII-1. Limitations of the available experimental techniques and perspectives for future research are discussed.

**Conclusions:**

The current review continues to emphasize the need for further, objective, evidence-based examination of the relationship between HHV-6 infection and multiple sclerosis.

## Introduction

**Fig. 1:**
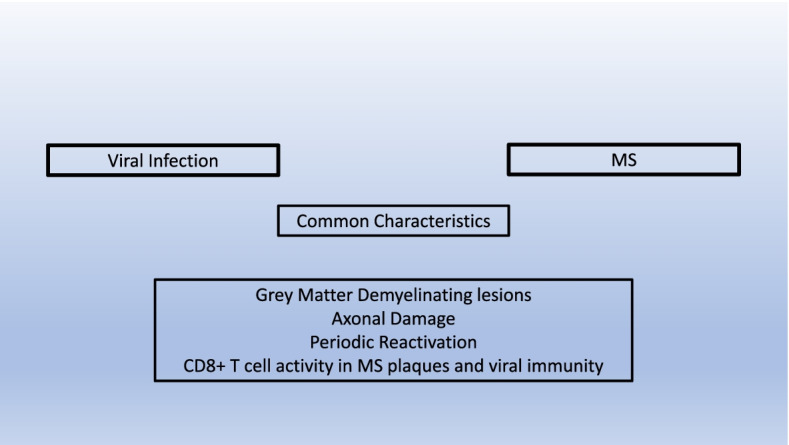
Common characteristics of a viral infection and demyelination in the form of Multiple Sclerosis

The role of viral agents in the pathogenesis of Multiple Sclerosis (MS) has been a matter of intense scientific interest [1]. Although to date, no specific viral agent has been implicated as a definite causative factor of MS, infections have long been thought to be significant contributors to disease development or exacerbation [2]. Results of familial aggregation and migration studies indicate that exposure to environmental factors, both infectious and non-infectious, during childhood and adult life are strong determinants of MS risk [3]. Thus, viruses remain at the epicenter of the research for MS pathogenesis for more than 40 years [2]. Figure 1 summarizes available data supporting a viral triggering effect on MS pathogenesis.

Among numerous viruses, Human herpesvirus 6 (HHV-6), an ubiquitous pathogen that falls into latency and periodically reactivates, represents a plausible candidate as a causative or triggering factor owing to its neurotropic, lymphotropic and immunomodulatory characteristics [3].Two variants of HHV-6 have been recognized: HHV-6A, with a more prominent preference for infecting neural cells, and HHV-6B [3, 4]. However, it is less often associated with disease than HHV-6B. HHV-6A has been found predominantly in MS lesions. Primary evidence backing a convincing pathogenic role for HHV-6 in MS was based on cerebrospinal fluid (CSF) detection of viral DNA by polymerase chain reaction (PCR). Observation of viral messenger RNA and protein expression in oligodendrocytes were additional contribution to the hypothesis of HHV-6 as an operator of MS [4]. In this review, an update is presented regarding the evidence on the potential relationship between HHV-6 and MS pathogenesis [4] as well as a possible alteration in the immunological status of MS HHV-6 seropositive patients versus MS seronegative ones.

## Methods

**Fig. 2:**
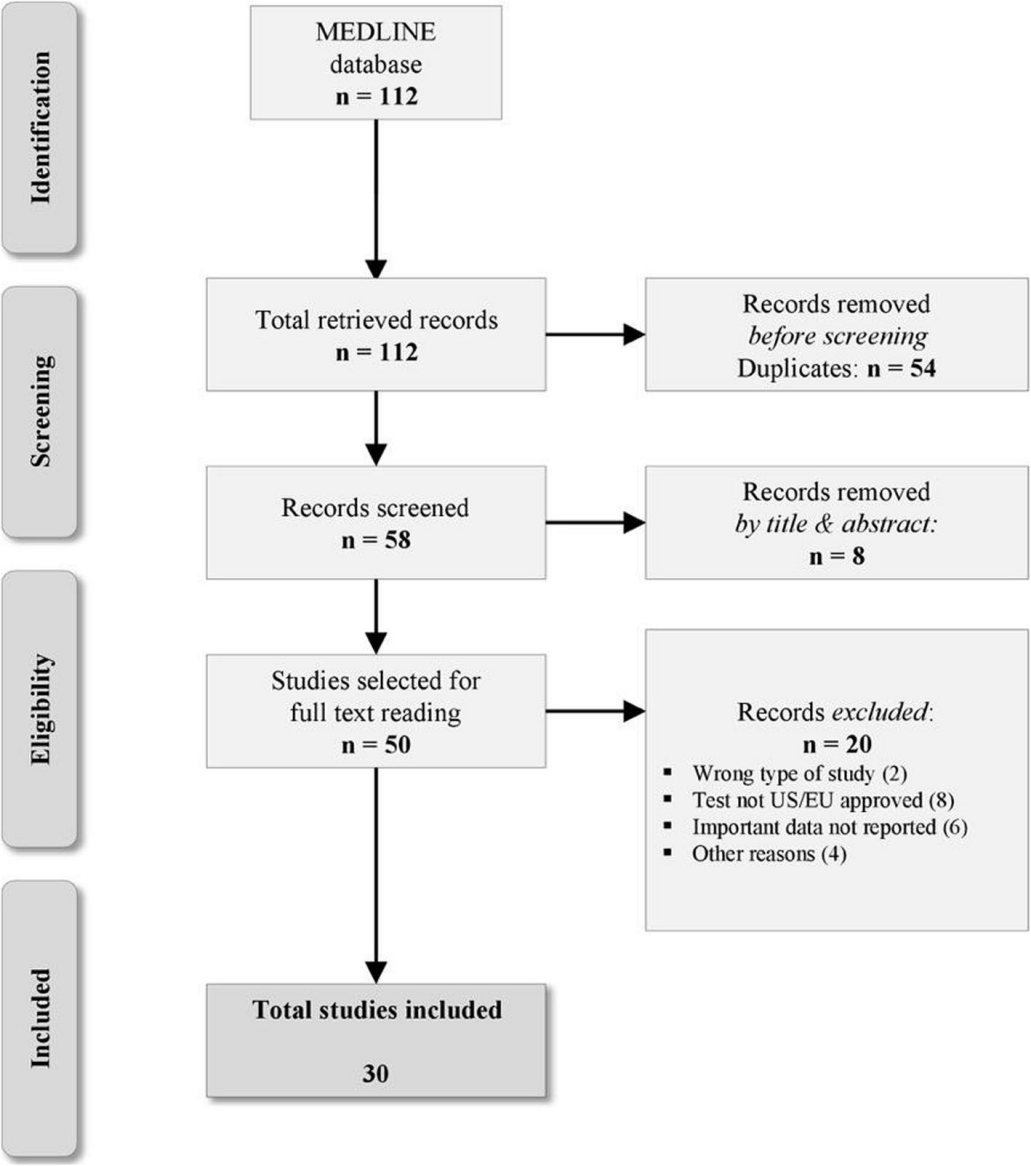
Study selection Flow Chart

We conducted a review of clinical studies reporting the detection of HHV-6 genome or anti-HHV-6 antibody response in patients with MS. The authors independently performed the literature search, study selection and data extraction. Studies reporting serological, cerebrospinal fluid (CSF) and genome studies identifying HHV-6 activity in MS patients were included while HHV-6 preclinical data and animal studies were excluded from the literature search. No language restrictions were applied to the literature search. Pubmed MEDLINE database was accessed covering the period from 01/01/2010 up to 30/06/2021 using the following search terms: human herpes virus 6, HHV-6, demyelination, multiplesclerosis, pathogenesis, diagnosis, serology, cerebrospinal fluid, CSF, IgG antibodies, IgM antibodies, PCR, polymerase chain reaction and polymorphisms.Retrieved studies from the initial search were further screened for additional articles. The quality of the included trials was classifiedaccording to the criteria proposed by Moore and Wolfson as well as those proposed by the Canadian Task Force (CTF) on the Periodic Health Examination [5, 6]. The studies with statistically significant correlation between MS and HHV-6 infection are presented in Table 1. The corresponding flow chart is presented on Fig. 2

**Table 1:** Studies with statistically significant correlation between MS and HHV-6 infection detected with serological, molecular and genotyping techniques

Study	Method	MS population	Additional Results	Moore and Wolfson	Canadian Task Force
Bistrom *et. al* (2021) [17]	Serum IgG/IgM	**670** individuals who later developed relapsing MS and 670 matched controls **HHV-6A seropositivity** was associated with increased MS risk in all age groups	Seropositivity against EBV exhibited a pattern where associations switched from a decreased risk of developing MS in the group below 20 years of age to an increased risk amongst individuals aged 20–29 and 30–39 years	A	II-1
Perlejewski *et al.* (2020) [37]	CSF PCR	4 MS patients and 13 controls Viral nucleic acid in **seven** (20.59%) MS patients and in one (7.69%) control patient	most frequently detected was human herpesvirus type 6 (HHV-6; **3 cases; 8.82%**);	A	II-1
Amini *et. al* (2020) [18]	Serum IgG/IgM	560 MS patients along with 560 healthy subjects were analyzed for HHV-6 seropositivity About **90.7%** of MS patients (508/560) were seropositive for HHV-6, while **82.3%** (461/560) of healthy subjects were seropositive for this virus (p = 0.001)	MMP-9 levels in seropositive MS patients were significantly higher than seronegative MS patients (***p* = 0.001**). EDSS in seropositive MS patients was significantly higher in comparison to seronegative MS patients (***p* < 0.05**)	A	II-1
Maria I Dominguez Mozo *et. al* (2020) [31]	Serum/CSF microRNA techniques	a significant correlation between the levels of serum hhv6b-miR-Ro6-2 and -3-5p, -2 and miR-U86 and -3-5p and miR-U86 also in the CSF, hhv6b-miR-Ro6-2 and -3-5p	The anti-HHV-6A/B IgG levels in CSF and the intrathecal antibody production in positive MS patients for hhv6b-miR-Ro6-3-5p **were statistically significant higher** than in the negative ones	A	II-1
Engdahl *et al*. (2019) [16]	Serum IgG/IgM	**8,742** MS and **478**patients with preclinical forms The IgG response against IE1A was positively associated with increased risk of future MS	The IgG response against IE1B was **negatively associated** with MS	A	II-1
Ortega-Madueno et al*.* (2014) [7]	Serum IgG/IgM	**69%** with a low HHV-6A/6B IgG titer after 2 years of DMTs free of relapse vs **40.7%** with an increased titer (higher significance for Natalizumab)	**HHV-6A IgG** titers peak **1 week** before relapse, **HHV-6A/6B IgM** titers peak **2 months** after relapse	A	II-1
Simpson S. et al*.* (2012) [12]	Serum IgG/IgM	Dose dependent trend between **rr** and **HHV6 IgG titer**	**Higher titers in female progressive MS patients** rather than male	A	II-1
Khaki M. et al*.* (2011) [14]	Serum IgG/IgM	61 patients and 60 controls screened for HHV6 amongst other viruses: **OR = 2 [95%CI1-4], *p* = 0.04**for **HHV6 IgG**	**OR4.3[95%CI:2–9.3], *p* = 0.001** between MS patients and controls for **HHV6 IgM**	A	II-1
Behzad-Behbahani *et al.* (2011) [15]	Serum IgG/IgM Serum PCR	**30%** serum samples with high HHV6 IgG titers and **33%**withpositive HHV6 DNA. Significantly higher prevalence of HHV6 DNA in MS patientsvs controls, ***p* = 0.001**	Higher HHV6 antibody and DNA titers in MS patients in relapse ***p* = 0.001**	B	II-1
Ben-Fredj *et al.*(2013) [11]	Serum IgG/IgM, Serum PCR	**51.47% U94/REP HHV6 protein** positive samples, **6.66%** HHV6-DNA positive samples	Statistically significant results in both antibody and PCR techniques for relapses vs remission	A	II-1
Moghadam et al*.* (2015) [19]	Serum PCR	28 out of 46 (**60.8**%) plasma samples of patients with MS were positive for viral DNA	The difference in prevalence of HHV-6 DNA in blood between patients with MS and controls was statistically significant **OR 0.277 [95%CI: 0.12–0.89], *p* = 0.001**	A	II-1
Garcia-Montojo M. et al*.* (2011) [25]	Serum PCR	HHV6 DNA detected more frequently in MS patients with relapse. The **rr** was higher in patients with HHV6 DNA in serum, as well as the severity of the relapse	HHV6 DNA and clinical parameters could not be associated The response to IFN treatment (measured by **rr** reduction and severity of the relapse) was significantly less in MS patients with HHV6 DNA in serum	A	II-1
Nora-Krukle Z. et al*.* (2011) [20]	Serum PCR	**57%**HHV6 DNA positive in RRMS patients **43%** HHV6 DNA positive in SPMS patients	**66%** patients without viremia were m-RNA HHV6 positive Significantly higher levels of IL-12 and TNF-a in MS patients with active infection vs patients with latent HHV6 infection	B	II-1
Dominguez-Mozo M et al. (2012) [24]	Genotyping, Serum PCR	**Low levels of MHC2TA m-RNA** levels at the **initial** visit and high**levelspostIFN** treatment in patients with active HHV6 infection vs patients without	High MHC2TA m-RNA levels post IFN treatment **58.6%** without active HHV6 infection responded to IFN treatment vs **23.8**% of patients with active HHV6 infection	A	II-1
Blanco-Kelly et al*.* (2011) [28]	Genotyping, serum PCR	Polymorphisms **TNFRSF6B** and **TNFRSF14** were associated with MS. The effects were stronger in patients with HHV6 active replication	TNFRSF6B-rs4809330**OR 1.13 [95%CI: 0.82–1.53], *p* = 0.0008** TNFRSF14-**rs6684865 OR1.2, *p* = 0.0008 [95%CI: 0.78–1.36]**	A	II-1
Garcia-Montojo M. et al*.* (2011) [25]	Genotyping, Serum PCR	MHC2TA polymorphisms correlated with active replication (**30.2%)** **Significant differences for MHC2TA between IFN-responders and non-responders**	**No significant results** between the groups when active replication wasnot included	A	II-1
Vandenbroeck K. et al*.* (2011) [26]	Genotyping, Serum PCR	Association of polymorphism IRF5-rs3807306T and HHV6 infection **OR 1.56 [95% Cl: 1.00–2.44], *p* = 0.05**	Response to IFN correlated with the same polymorphism (not statistically significant) **OR 1.39 [95% Cl: 0.95–2.05], *p* = 0.09**	A	II-1
Alvarez-Lafuente R. et al*.* (2010) [27]	Genotyping,Serum PCR	**Association of MHC2TA-rs4774C with HHV6 active replication**	**Allele C** correlated with a different clinical response to treatment for MS patients **33%** of MS patients without the allele responded to IFN, ***p* = 0.05**, **31%** of MS patients without the allele had a better disease progression (**EDSS = 1**), ***p* = 0.01**	A	II-1
Alenda R et al*.*(2014) [32]	CSF IgG/IgM	**Presence of intrathecal HHV6 IgG antibodies** in the CSF of MS patients	Major antigen is the major HHV6 capsid protein	A	II-1
Pietiläinen-Nicklén J et al*.* (2014) [33]	CSF IgG/IgM	**Presence** of HHV6 OCBs in MS patients	**Earlier manifestation** of the diseases in HHV6OCB patients	A	II-1
Virtanen JO et al*.* (2014) [35]	CSF IgG/IgM	**38%** of the MS patients had OCBs related to HHV6	**HHV6 OCBs correlated** with more GdE ( +) lesions	A	II-1
Virtanen JO et al*.* (2011) [36]	CSF IgG/IgM	**18%** of OCB( +)ve patients were HHV6( +)ve,**26%**of the OCB( +)ve MS patients had HHV6 OCBs	HHV6( +)ve OCBs in other demyelinating diseases (**21%**) and other neurological diseases (**10%**)	A	II-1
Pietiläinen-Nicklén J*et al* (2010) [34]	CSF IgG/IgM	**Presence of OCBs** in both acute and chronic MS	**Association** of the HHV6 infection with the symptoms of clinically possible MS	B	II-1

*CI* Confidence interval, *CSF* Cerebrospinal fluid, *DMTs* Disease modifying therapies, *GdE ( +)* Gadolinium enhancing, *HHV6* Human Herpesvirus 6, *IFN* Interferon, *Ig* Immunoglubulin, *IL-12* Interleukin 12, *m-RNA* messenger RNA, *MS* Multiple Sclerosis, *OCBs* Oligoclonal bands, *OR* Odds ratio, *PCR* Polymerase chain reaction, *rr* annual relapse rate, *RRMS* Relapsing remitting Multiple Sclerosis, *SPMS* Secondary progressive MS, *TNF-a* Tumor necrosis factor alpha, *MMP-9* Matrix Metalloproteinase 9, *EDSS* Expanded Disability Status Scale

## Results

### Serological Studies

In total, **9** studies reported the measurement of serum anti-HHV-6 IgG and/or IgM antibodies, in their methodology. Among these studies, **8 **concluded on a statistically significant correlation between quantitative HHV-6 serology results and MS evolution as presented in Table 1. As an example, Ortega-Madueño *et al. *found that 69% of MS patients who were free of relapses and disease progressionhadlow anti-HHV-6A/B IgG titers after 2 years of different Disease Modifying Therapies (DMTs) compared to 40.7%with an increase of antibody titers (*p* < 0.001); the highest significance was notedin patients who received Natalizumab [7]. Natalizumab is an immune modulator that impairs the entry of B and plasma cells into the central nervous system (CNS) and, therefore, is associated with decreased total levels of IgG and IgM in CSF [8–10]. Additionally, Ortega-Madueño *et al. *found a peak ofanti-HHV-6A/B IgG and IgM titerstwo weeks and one-month, respectively, prior tothe occurrence of a clinical relapse [7]. Another study showed that the IgG antibodies against the latency associated HHV-6 protein (anti-HHV-6 U94/REP IgG) were higher in MS patients compared to healthy controls. In addition, a significant difference in anti-HHV-6 U94/REP immunoglobulin titers was depicted between the relapse and remission phases [11]. Similarly, a prospective cohort study demonstrated that anti-HHV-6 IgG titer was positively associated with the hazard of disease relapse with a dose-dependent trend [12]. However, the same authors found that this association was not mediated by the reactivation of HHV-6, as neither anti-HHV-6 IgM nor HHV-6 viral load were associated with relapse hazard, disability, or disability progressionin a subsequent study [13]. Another team that screened MS patients and controls for antibodies against numerousviral pathogens, found significantly higher seroprevalence of both anti-HHV-6 IgG and IgM in MS patients compared to healthy controls [14]. Likewise, Behzad-Behbahani *et al*. reported higher anti-HHV-6 titers in MS patients’ sera, especially during a clinical relapse [15]. Engdahl *et al.*found that the IgG response against the immediate-early 1 (IE1) protein of HHV-6 A was positively associated with MS [odds ratio (OR) = 1.55, *p* = 9 × 10^−22^], as well as with an increased risk for future development of MS (OR = 2.22, *p* = 2 × 10^−5^) [16]. In addition, Bistrom *et al.* found that HHV-6A was associated with increased MS risk in all age groups (total cohort odds ratio 2.1, 95% confidence interval 1.6–2.7) [17] while Amini *et al*. demonstrated a significant HHV-6 seropositivity (90.6% in 560 MS patients) and significantly higher levels of Matrix Metalloproteinase 9 (MMP-9) in HHV-6 seropositive patients (p = 0.001) as well as Expanded Disability Status Scale (EDSS) score [18].

As evidenced above, most studies show a significant difference in anti-HHV-6 antibody titers in MS patients compared to controls, significantly higher inflammatory markers higher EDSS disability scores, in more recent studies, and higher titers during clinical relapses. The main issue with the existing trials is the lack of a uniformly accepted positive titer threshold. Hence, positive results are interpreted based ondiverse criteria among studies. Moreover, seropositivity doesnot necessarily correlate with lymphoproliferation, as positive HHV-6 antibodies are also detected in healthy controls. Finally, the interpretation of the positive IgM antibodies remains ambiguous, and may represent either recent infection or viral reactivation or even a latent or persistent subclinical infection which can be uncovered under certain circumstances, like immunosuppression.

### Polymerase Chain Reaction techniques

The presence of HHV-6 genome in patients with MS was investigated in **14 **studies using Polymerase Chain Reaction (PCR) techniques. Studies performing genotyping techniques are described separately. Among those, **12 **found a positive correlation between the presence of viral nucleic acid and MS (Table 1). One study found that HHV-6 genomic sequences werepresentin 28 (60.8%) out of 46 plasma samples taken from MS patients, compared to 13 (28.2%) out of 46 positive samples in the control group (OR = 0.277, *p* = 0.0027) [19]. Similarly, another two studies performing both serologic and PCR techniques*,* reported higher prevalence of HHV-6 deoxyribonucleic acid (DNA) in MS patients compared to controls [11, 15]. Additionally, a small study detected HHV-6 DNA in 4 out of 7 plasma samples of taken from patients with relapsing–remitting MS (RRMS) and in 4 out of 7 patients with secondary progressive MS (SPMS), during disease exacerbation, but not during remission; even though plasma viremia was undetectable in the remaining 3 RRMS patients, viral messenger ribonucleic acid (mRNA) transcription was detected in 2 of them [20]. In another study, the authors demonstrated that patients with detectable HHV‐6 viremia had a higher risk of severe relapses and a worse response to Interferon beta (IFN-beta) therapy. Moreover, HHV‐6 was detected more frequently during relapses, while patients with detectable HHV‐6 in their sera had more frequent relapses, lower reduction of the relapse rate, and decreased proportion of responders to IFN-beta compared to those without an active viral replication [21]. Besides the studies reporting the prevalence of HHV-6 in blood and serum samples, the virus has been also isolated in urine samples fromMS patients. Indeed, Esmaili *et al.* reported a prevalence of HHV-6 DNA of 13.8% (23 out of 60) in urine samples obtained from patients with MS, whereas none of the 60 urine specimens from healthy controls was tested positive[22].

An important aspect which should be taken into account when investigating the detection of HHV-6 in blood or serum with PCR techniques is the phenomenon of chromosomal integration of human HHV-6 (ciHHV-6). This is a condition whereby the complete HHV-6 genome is integrated into the host’s germ line genome and is vertically transmitted in a Mendelian manner [23]. The condition is found in less than 1% of controls in the USA and the United Kingdom. HHV-6 levels in whole blood that exceed 5.5 log10 copies/ml are strongly suggestive of ciHHV-6 [23]. Monitoring DNA load in plasma and serum is unreliable, both for identifying and for monitoring subjects with ciHHV-6 due to cell lysis and release of cellular DNA. High HHV-6 DNA loads associated with ciHHV-6 can lead to erroneous diagnosis of active infection [23].

Regarding studies performing genotyping techniques, Dominguez-Mozo *et al. *found that active replication of HHV-6 decreases the MHC2TA gene mRNA levels, a gene that plays a key role in controlling the immune response against viruses,and that low levels of serum HHV-6 DNA predict better response to IFN-beta treatment [24]. Likewise, Garcia-Montojo *et al. *demonstrated that MHC2TA polymorphisms correlated with active viral replication (30.2%), while there were significant differences for MHC2TA between IFN-beta responders and non-responders in MS patients [25]. Two additional studies found a correlation between two frequently encountered gene polymorphisms in MS patients (IRF5-rs3807306T and MHC2TA-rs 7447C), with active HHV-6 replication [26, 27]. Both, correlated HHV-6 active replication with low IFN-beta responsiveness. Another trial showed that TNFRSF6B-rs4809330(*)A and TNFRSF14-rs6684865(*)A gene polymorphisms were associated with MS predisposition, and especially in patients with active HHV-6 replication [28].

Latham *et al*. found that the increased antiviral immune responses, directed primarily againstthe Epstein-Barr virus (EBV) virus, were significantly correlated with subsequent disease activity on Magnetic Resonance Imaging (MRI) scan in the form of Combined Unique Active lesions (CUAl). Regarding the HHV-6 the results were non-significant results [29]. Also, Ferrante *et al*. found a high frequency of HHV-6 DNA detection in peripheral blood mononuclear cells (PBMCs) isolated not only from patients with acute (41.6%) and stable (22.2%) MS, but also from healthy controls (45.9%), without depicting a statistically significant difference among the three groups [30].

In the first study of its kind in the field of MS and HHV-6, Mozo and colleagues analyzed the micro-RNAs of HHV-6A: in paired samples of serum and CSF of 42 untreated MS patients and 23 patients with other neurological diseases using MicroRNA Assay techniques. Intrathecal HHV-6A/B antibody production and anti-HHV-6A/B IgG/IgM levels in serum were measured. In the serum of the whole population (MS and OND patients) they found a significant correlation between the levels of hhv6b-miR-Ro6-2 and -3-5p (Spearman *r* = 0.839, pcorr = 3E-13), -2 and miR-U86 (Spearman *r* = 0.578, pcorr = 0.001) and -3-5p and miR-U86 (Spearman *r* = 0.698, pcorr = 1.34E-5); also in the CSF, between hhv6b-miR-Ro6-2 and -3-5p (Spearman *r* = 0.626, pcorr = 8.52E-4). The anti-HHV-6A/B IgG levels in CSF and the intrathecal antibody production in positive MS patients for hhv6b-miR-Ro6-3-5p were statistical significantly higher than in the negative ones (pcorr = 0.006 and pcorr = 0.036) [31].

The above mentioned studies attempted to investigate the presence and the effects of HHV-6 active replication to the clinical course of MS as well as the responsiveness of the disease to IFN-beta treatment. All studies found an inverse correlation between HHV-6 replication and the patients’ response to IFN-beta, which is probably suggesting that HHV-6 DNA may play a predictive role in treatment responsiveness and implying a possible association of viral active replication with the presence of IFN-beta neutralizing antibodies. The significance of the detectable viral DNA remains unclear, as reactivation does not always imply clinical infection. As such, these results should be interpreted cautiously.

### Cerebrospinal fluid studies

The detection of anti-HHV-6 antibodies, oligoclonal bands (OCBs) and/or HHV-6 DNA in CSFwere investigated in **7 **studies; all of them found a positive correlation between the presence of HHV-6 and MS and reported the presence of intrathecal HHV-6 OCBs in MS patients [32–36]. The frequency of positive CSF samples ranged between 18 and 38%, while, the detection of HHV-6 DNA in the CSF was positively correlated with the amount of gadolinium enhanced (GdE +) lesions in the MRI [32–36]. In a more recent HHV-6 DNA detection study Perlejewski *et al.* searched for viral RNA and DNA in the CSFof 34 MS patients and 13 controls using RT-PCR/PCR. It revealed the presence of viral nucleic acid in seven (20.59%) MS patients and in one (7.69%) control patient. In MS patients the most frequently detected was human herpesvirus type 6 (HHV-6; 3 cases; 8.82%); followed by Epstein-Barr virus (EBV; 2 cases; 5.88%), varicella zoster virus (VZV; 1 case; 2.94%) and Enterovirus (EV; 1 case; 2.94%) [37].

Both IgG and intrathecal production and OCBs can be detected in the CSF of MS patients. However, the major difference between themis that intrathecal IgG production is polyclonal while OCBs are a marker of clonal antigen-specific activation. Thus, OCBs allow the identification of a disease relevant antigen [32]. Consequently, it has been attempted to correlate HHV-6 with the formation of OCBs in MS.An equally interesting theory is that HHV-6 OCBs remain the same over the course of MS. Previous data suggested that MS patients with HHV-6 or EBV OCBs had fewer GdE + lesions [38]. One study demonstrated that HHV-6 DNA in CSF was associated with the number of GdE + lesions, invigorating an interesting hypothesis that HHV-6 OCBs in CSF control the viral activity within the CNS and limit the damage to brain tissue due to viral activation within the CNS [35]. This hypothesis is consistent with the findings that both HHV-6 antibody titers and DNA levels are higher in serum during a clinical relapse [15]. However, one must bear in mind that in many cases CSF studies are performed based on the availability of the CSF samples. Consequently, it is quite burdensome, or even impossible, to achieve a careful cross match between MS and control samples.

## Discussion

The vast majority (**27 out of 30**) of the studies presented in this review concluded on a significant association between HHV-6 detection and various clinical aspects of MS, implying a possible pathogenetic linkage between them. However, there is a different interpretation of the same findings as presented above.

A breakdown in the Blood Brain Barrier (BBB) during an acute infection with HHV-6, may result in the same antibody detection in sera or CSF samples. In both cases the antibodies will originate from the periphery which, of course, does not suggest a direct implication of HHV-6 in the pathogenesis of MS. In addition, there are many alternative scenarios that theoretically may lead to the presence of HHV-6 antibodies in the biological fluids of MS patients. Some of those are reactivation of a latent HHV-6 infection, a subclinical infectious course without any clinical symptoms to i.e. immunocompromised patients and immune system hyperactivity in the case of MS relapse. Moreover, a positive sample for HHV-6 antibodies can be the result of an infection in childhood or an active infection in adult life. It remains to be seen whether these two conditions can have the same influence in pathogenesis of MS, given the fact that their chronologic encounter is quite different. Conclusively, HHV-6 antibodies studies highlight the importance of a positive sample no matter what the mechanism behind this positivity is.

PCR techniques can give an answer to the previous questions given the fact that they can identify actual viral replication. However, PCR HHV-6 specific techniques cannot distinguish between an active new infection from the reactivation of a latent disease or a chromosomal integration which requires special tissue testing. It remains to be answered whether these three conditions may cause the same immune hyperactivity and more pronounced neurological symptoms. The quantification of the exact viral load may shed some light to this problem as active infection is associated with higher viral load. In addition, it may contribute to the clarification of whether reactivation of a latent infection or chromosomal integration may have the same impact as an active new infection to the pathogenesis of MS.

Moreover, there are many alternative scenarios that theoretically may lead to the presence of HHV-6 antibodies in the biological fluids of MS patients. Some of those are reactivation of a latent HHV-6 infection, a subclinical infectious course without any clinical symptoms but to i.e. immunocompromised patients and immune system hyperactivity in the case of MS relapse.

In addition, a positive sample for HHV-6 antibodies can be the result of an infection in childhood or an active infection in adult life. It remains to be seen whether these two conditions can have the same influence in pathogenesis of MS given the fact that their chronologic encounter is very different.

Ultimately and in order to tackle with the above conditions and alternative interpretations of the same results, a controlled clinical trial of an efficacious and CNS penetrable anti HHV-6 drug may be the only way to ascertain the actual role of HHV-6 to MS. The authors think that robust clinical results in favor of a positive impact on the clinical, radiological and less likely disability parameters of MS would contribute to the adaptation of a significant environmental MS pathogenetic role while ambiguous or negative results will add to the existing controversy.

In any case, the authors think that there must be certain conditions fulfilled so as to facilitate a solid trial investigating the pathogenetic role of HHV-6.

A. The controls used should be as similar to MS patients: Whenever possible the researchers have to ensure that the only difference between the research group and the control group is the well-established diagnosis of MS. Apart from the apparent age, sex and race cross match it is imperative for the control participants to come from the same population pool as the MS group participants. This is to ensure that the genetic background especially regarding the various polymorphisms related with MS that in many cases are population specific will be the same for the two groups. As a result, the interactions between environmental factors (HHV-6) and the genome (samples from the same population and consequently same prevalence of polymorphisms related to MS) wont influence the possible HHV-6 role to MS.

B. Participants in the MS group have to have well-defined diagnostic criteria and ideally to be matched according to disease onset. A research group consisted of MS patients with robust diagnostic criteria and as much as possible similar disease onset, which means similar disease profile and clinical course, will ensure investigating the possible role of HHV-6 on the same substrate, where similar pathogenetic mechanisms take place. Inclusion of patients with progressive forms of the disease for example, does not necessary implicate the presence of the virus, in the case of a positive result, during the time of diagnosis or that its presence was more than a chance event.

C. Universal HHV-6 sample positivity threshold has to be established so as the research criteria to be the same in all studies. It is also essential to present all negative samples as well to rule out the possibility of a group of negative controls due to an experimental role. Finally, it is preferred that the results to be evaluated by observers blinded to the diagnosis.

Two important aspects regarding the HHV-6 is the HHV-6 biology and the HHV-6 interactions with the genome.

Another aspect that needs to be noted is based on the study of Leibovitch *et al. *which proved that HHV-6A DNA is more prevalent than HHV-6B in saliva samples of MS patients vs controls. This is probably because HHV-6A is more neurotropic than HHV-6B [39]. As a consequence, the authors think that it is important to fully investigate the properties of every virus and elucidate their effect on the CNS. Hence, all future studies have to distinguish between the various types of HHV-6 as they seem to exhibit different behavior. This may cause different antibody reactivity, behavior and even time of acquisition of HHV-6. Different types of HHV-6 may mean different time threshold of a positive sample.

Interaction of the HHV-6 with the genome is also a very promising field in the HHV-6 pathogenetic research. In every study there is a correlation with MS related polymorphisms or genes. Is this perhaps due to specific areas in the HHV-6 genome in MS patients? And if that is the case are there different genome areas in HHV-6 DNA in MS patients and controls? Or is this location specific? Different HHV-6 genome sequence in CNS located HHV-6 and in HHV-6 located in the periphery? After all we are discussing about a virus with ubiquitous nature which is hypothesized to be implicated in a non-ubiquitous inflammatory pathogenetic mechanism. Perhaps the reason behind this paradox is genetic variability of HHV-6.

The evidence regarding the pathogenetic role of HHV-6 is solid and engulfs several different methodological approaches. Future research needs to be designed to answer all the methodological and pathophysiological queries that have aroused based on the existing data. HHV-6 antiviral medication trials will also contribute immensely in the clarification of the nature of HHV-6’s role in the pathogenesis of MS.

## Data Availability

The datasets used and/or analyzed during the current study available from the corresponding author on reasonable request.

## Abbreviations

BBB: Blood Brain Barrier
ciHHV-6: Chromosomal integration of HHV-6
CNS: Central Nervous System
CSF: Cerebrospinal Fluid
DMT: Disease Modifying Therapy
DNA: Deoxyribonucleic acid
EBV: Epstein-Barr Virus
EDSS: Expanded Disability Status Scale
GdE+: Gadolinium Enhanced Lesion
HHV-6: Human Herpes Virus 6
IFN-Beta: Interferon Beta
MMP-9: Matrix Metalloproteinase 9
MRI: Magnetic Resonance Imaging
mRNA: RNA messenger
MS: Multiple Sclerosis
OCB: Oligoclonal Bands
OR: Odds Ratio
PCR: Polymerase Chain Reaction
RNA: Ribonucleic acid
